# Classification of Highly Divergent Viruses from DNA/RNA Sequence Using Transformer-Based Models

**DOI:** 10.3390/biomedicines11051323

**Published:** 2023-04-28

**Authors:** Tariq Sadad, Raja Atif Aurangzeb, Mejdl Safran, Imran, Sultan Alfarhood, Jungsuk Kim

**Affiliations:** 1 Department of Computer Science, University of Engineering & Technology, Mardan 23200, Pakistan; 2 Department of Computer Science & Software Engineering, International Islamic University Islamabad, Islamabad 44000, Pakistan; 3 Department of Computer Science, College of Computer and Information Sciences, King Saud University, Riyadh 11543, Saudi Arabia; mejdl@ksu.edu.sa (M.S.);; 4 Department of Biomedical Engineering, Gachon University, Incheon 21936, Republic of Korea; 5 Department of Biomedical Engineering, Gachon University, Seongnam-si 13120, Republic of Korea

**Keywords:** BERT, deep learning, DNA/RNA sequence, K-MERS

## Abstract

Viruses infect millions of people worldwide each year, and some can lead to cancer or increase the risk of cancer. As viruses have highly mutable genomes, new viruses may emerge in the future, such as COVID-19 and influenza. Traditional virology relies on predefined rules to identify viruses, but new viruses may be completely or partially divergent from the reference genome, rendering statistical methods and similarity calculations insufficient for all genome sequences. Identifying DNA/RNA-based viral sequences is a crucial step in differentiating different types of lethal pathogens, including their variants and strains. While various tools in bioinformatics can align them, expert biologists are required to interpret the results. Computational virology is a scientific field that studies viruses, their origins, and drug discovery, where machine learning plays a crucial role in extracting domain- and task-specific features to tackle this challenge. This paper proposes a genome analysis system that uses advanced deep learning to identify dozens of viruses. The system uses nucleotide sequences from the NCBI GenBank database and a BERT tokenizer to extract features from the sequences by breaking them down into tokens. We also generated synthetic data for viruses with small sample sizes. The proposed system has two components: a scratch BERT architecture specifically designed for DNA analysis, which is used to learn the next codons unsupervised, and a classifier that identifies important features and understands the relationship between genotype and phenotype. Our system achieved an accuracy of 97.69% in identifying viral sequences.

## 1. Introduction

Human beings and mammals can be infected by viruses that can spread easily through contact with saliva, blood, or even through sneezing. Viruses have the ability to mutate into different variants [1] and strains, which can potentially make vaccines ineffective. Therefore, early and cost-effective diagnosis is crucial for preventing the spread of viruses and reducing mortality rates. For instance, HCV is an RNA virus that infects human liver cells, and chronic HCV infection can lead to a range of liver diseases, including hepatitis, liver fibrosis, cirrhosis, and liver cancer. The early detection of HCV is important as there is currently no known cure or vaccine for the virus. If left untreated, HCV can cause severe liver damage and increase the risk of liver cancer [2]. Additionally, mononucleosis, also known as the “kissing disease”, has been linked to several types of cancers, including Burkitt’s lymphoma, Hodgkin’s disease, and nasopharyngeal carcinoma. Similarly, HBV is an example of an oncovirus that can cause genomic instability, which can lead to the development of hepatocellular carcinoma, the fifth most common cancer worldwide. Another example is human papillomavirus (HPV), a double-stranded, circular DNA virus that can cause various epithelial lesions and cancers, including cutaneous and anogenital warts that may progress to carcinoma depending on the subtype. Polymerase Chain Reaction (PCR) is a widely used technique to amplify and detect specific nucleic acid sequences from various sources, including viral particles in blood samples. The resulting DNA sequences obtained from PCR can be used to identify viruses, their strains, and variants. These sequences can be compared to reference databases, such as the NCBI GenBank, to identify the presence of viral genetic material and determine the closest matches to known viral sequences [3].

BLAST (Basic Local Alignment Search) is a widely used bioinformatics tool that compares DNA or protein sequences against a database to find similar sequences [4]. However, just finding similarities between the collected genome and a reference genome using BLAST is not always sufficient to identify a pathogen, as there may be other factors to consider. Some biological features, such as the presence of DNA-binding proteins, can accurately and quickly predict the presence of viruses. Deep learning algorithms can be used to classify DNA based on these features and provide more accurate predictions [5].

This paper aims to explore computational methods to detect viral genomes and predict integration sites to understand the organs most affected by viral infections. This information can help to develop targeted treatments for viral infections and improve patient outcomes.

## 2. Literature Review

Computational virology has witnessed notable progress in recent years, with the widespread application of machine learning (ML) and deep learning (DL) techniques [6], for DNA classification and virus identification [7]. In one study [8], ML algorithms were compared with and without feature extraction for DNA classification, and the authors concluded that ML could be used to investigate the origin of SARS-CoV-2 viruses. Another study [9] analyzed DNA sequence classification using convolutional neural networks (CNN) and hybrid models, limited to coronaviruses, dengue, hepadna-viruses, and influenza. The study found that deep neural networks could predict the host directly from genome sequences, but highlighted the limitations of LSTM gradient accumulation issues over large nucleotide sequences and generalization problems due to a lack of complex adaptation features to identify the host. Similarly, in [10], the authors proposed deep learning for viral host prediction, evaluating the effectiveness of deep neural networks on influenza A, rabies lyssaviruses, and rotavirus using the European Nucleotide Archive (ENA) database. However, the use of long nucleotide sequences can pose a challenge for the deep neural network, as it faces LSTM gradient accumulation issues. Additionally, there may be a generalization problem with the model, as it may lack the necessary complex adaptation features to accurately identify the host from genome sequences. SVMs and regression models were presented in another study [11] that focused on novel viruses without taxonomic assignment, but they required long input sequences and only broad host categories were supported. A transformer model based on the BERT architecture [12] was proposed in [13] for eukaryotic, bacterial, archaeal, and viral sequences, relying on natural language processing and bidirectional encoding. However, the prediction accuracy was lower for the lowest taxonomic rank (genus). In [14], a LSTM model was used for DNA classification, and the study focused on prokaryotic genomes. For eukaryotes, a classifier was proposed to distinguish between coding and noncoding DNA and predict reading frames for only the CDS (coding sequences). In a study conducted by [15], a DL architecture was proposed to predict short sequences in 16s ribosomal DNA, resulting in a maximum accuracy of 81.1%. Another study [16] proposed a spectral-sequence-representation-based deep learning neural network, which was tested on a dataset of 3000 16S genes and compared with GRAN (General Regression Neural Network). The study found that better results were obtained by optimizing the model’s hyperparameters. Furthermore, the importance of big data in intelligent learning was emphasized in [17]. The authors in [18] used machine learning and deep learning techniques in virus identification and DNA classification to treat COVID-19 patients, and achieved good results. However, there are also challenges associated with the employment of these techniques, such as LSTM gradient accumulation issues, generalization problems, and computational cost. These challenges need to be addressed through continued research and development to improve the accuracy and applicability of these approaches in understanding and combatting viruses.

To summarize, these studies demonstrate the potential of machine learning and deep learning techniques for virus identification and DNA classification. However, they also highlight the challenges associated with these techniques, including LSTM gradient accumulation issues, generalization problems, and the computational cost of feature selection. Despite these challenges, the use of deep neural networks in predicting host identification from genome sequences shows promise for the future of computational virology. With continued research and development, machine learning and deep learning techniques can aid in the identification and classification of viruses, potentially leading to better diagnosis, treatment, and prevention of viral diseases. However, some research gaps have been identified in the classification of various types of DNA sequencing for diseases using a generalized model. To address this gap, this study presents a novel deep learning model for the classification of various diseases such as Zika, influenza, HPV, WNA, hepatitis, and dengue, and the majority of this research consists of two main components.

- The first component involves a pipeline for nucleotide acquisition using the NCBI GenBank database to train a BERT tokenizer.
- The second component is specialized BERT architecture for DNA analysis that learns unsupervised next codons and passes the last hidden state of the CLS token to a classifier to identify relevant features for understanding genotype–phenotype relationships.

## 3. Materials and Methods

Our proposed method for analyzing viral genomic data is based on advanced natural language processing (NLP) [19] techniques, with a focus on developing a specialized BERT tokenizer that can extract relevant features from nucleotide sequences. The method comprises two main components: a basic pipeline for nucleotide acquisition and a specialized BERT model for genomic data analysis.

The first component involves collecting nucleotide sequences of different viruses from the NCBI GenBank database, which are then used to train the BERT tokenizer. This tokenizer breaks down the nucleotide sequences into smaller units called tokens, with each token consisting of three possible nucleotide combinations or codons.

The second component of the proposed system is a scratch BERT architecture designed specifically for DNA analysis. This architecture learns the next codons in an unsupervised manner, and then the last hidden state of the CLS token is passed to a classifier. The classifier identifies relevant features that are crucial for understanding the relationship between genotype and phenotype [20].

Our proposed method is particularly suitable for addressing the challenges associated with analyzing long genome sequences, which require significant computational power. Language transformer models, such as BERT, are particularly effective for this task because they can learn complex patterns and relationships from genome sequences. Unlike traditional machine learning methods, these models can extract more meaningful and complex features from the data.

### 3.1. Dataset

The dataset utilized in this study was obtained from GenBank [3], a publicly available open-source database that provides access to the latest nucleotide sequences for the research community. The employed dataset includes genome sequences of various viruses, such as *IAV*, *IBV*, *ICV*, *SFTS*, *Dengue*, *EnteroA*, *EnteroB*, *HBV*, *HCV*, *HSV-1*, *HPV*, *MPV*, *WNV*, and *Zika*.

Table 1 provides an overview of the different viruses included in the dataset, which are classified into different taxonomic levels including order, family, genus, and species. The Order column groups viruses with similar functions or characteristics. The Species column is the lowest level of classification, and groups viruses that share genetic and biological characteristics. The table lists various species of viruses, and this paper focuses on their classification.

### 3.2. Basic Pipeline for Nucleotide Sequence Acquisition 

**Figure 1:**
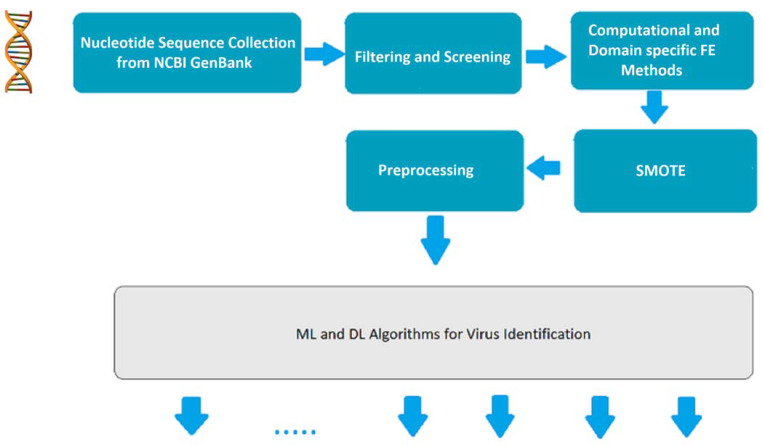
Basic architecture of the system pipeline.

Due to the lack of publicly available datasets containing nucleotide sequences for large sets of viruses, we have opted to collect the genomes of various viruses from the NCBI GenBank databases [3]. As the genome sequences are in a raw and heterogeneous format, it is essential to develop a robust and state-of-the-art BERT architecture. Figure 1 illustrates the fundamental architecture of the pipeline system, which includes the following components.

#### 3.2.1. Nucleotide Sequence Collection from GenBank

The first stage of the system pipeline entails the collection of viral nucleotides. This crucial step involves the thorough examination of publicly accessible nucleotide databases that are specifically designed to serve the research community. Through this initial stage, the system can effectively gather the necessary data required to proceed with subsequent analysis and processing.

#### 3.2.2. Filter and Screening

To ensure the quality and relevance of genomic data, a Python script was employed to filter raw and heterogeneous nucleotide sequences. This step was necessary to isolate the relevant data from the sequences. Additionally, given that the analysis was conducted solely in the Homo sapiens host cell, the host cell was also filtered to eliminate any extraneous data. Through this rigorous filtering process, the resulting dataset was optimized for subsequent analysis and interpretation.

#### 3.2.3. K-Mers for Computational and Domain-Specific Feature Extraction

Following the collection of genomic data, the genomes were transformed into k-mers [21], which are sets of possible nucleotide sequences of size k. This approach enables the identification of hidden patterns in DNA/RNA sequences. Subsequently, the BERT tokenizer was trained on the k-mers to generate DNA-specific tokens, which were utilized in the proposed BERT model. Through this process, the resulting model was optimized for the accurate analysis and interpretation of genomic data.

#### 3.2.4. SMOTE

In the context of disease-related data, imbalanced genomic data samples are a common occurrence, particularly with rare viruses such as *ZIKA*, *MPV*, and *WNV*, which may have divergent genotypes and very few nucleotide sequences available in databases. To address this issue, synthetic data can be added to the minority classes using SMOTE (synthetic minority over-sampling technique) [17]. This approach generates new samples to balance the data samples, in contrast to under-sampling techniques. By oversampling using SMOTE, the bias often exhibited by deep learning models towards majority classes in unbalanced datasets can be mitigated. Thus, SMOTE is a valuable approach in building deep learning models that are not biased towards the majority classes.

#### 3.2.5. Additional Preprocessing

It is essential to note that each deep learning or machine learning algorithm requires distinct types of preprocessing stages. These stages are necessary to ensure that the input data are optimized for subsequent analysis and interpretation. Specifically, preprocessing for the proposed BERT model will be expounded upon in the subsequent section.

### 3.3. Proposed BERT Model

**Figure 2:**
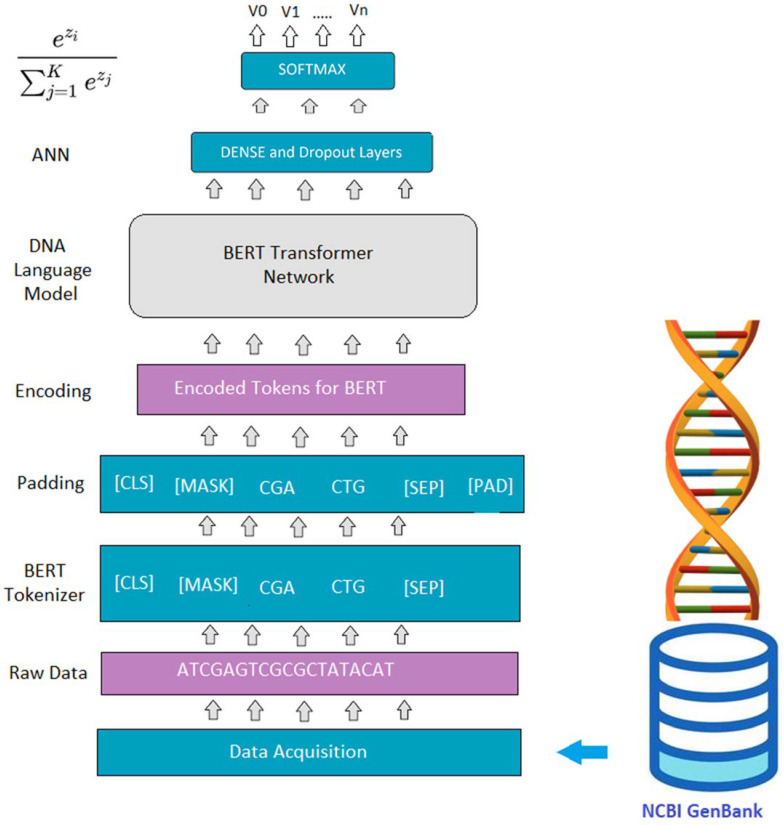
Proposed BERT Architecture.

The proposed BERT model [12] comprises transformer-based building blocks as presented in Figure 2, each of which serves a distinct purpose in the pipeline. The stages of the pipeline are explained below:

#### 3.3.1. Proposed DNA/RNA Tokenizer

In this stage, the nucleotide sequence is pre-processed for the custom BERT model, which has been trained on thousands of nucleotides. As the BERT Tokenizer is trained on Wikipedia data, a pre-trained BERT model was not used. Instead, the BERT Tokenizer was trained for genome data using various K-MERS parameters to optimize the BERT architecture.

#### 3.3.2. BERT Padding

Given that the length of 3-mers varies from sequence to sequence, and the maximum length of nucleotide sequences is 7000 bp, any gaps or missing sequence regions were padded with specific tokens. This ensured that the input sequence had a fixed length for subsequent analysis.

#### 3.3.3. Bidirectional Encoder Representation

The BERT model follows a specific format for training on K-MERS strings [22]. The input K-MERS are encoded into a bidirectional representation by the encoder. BERT can extract specific biomarkers from the genome in an unsupervised manner, and we can then pass these biomarkers into a deep neural network-based classifier. This stage of the pipeline is crucial for accurately analyzing and interpreting genomic data using the proposed BERT model.

#### 3.3.4. Classifier

In the field of machine learning, classifiers are composed of various possible sets of layers, such as dropout, ReLU, and softmax, among others. The selection of the optimal set of layers is determined through experiments performed with different parameters. Once attention-based features have been extracted, they are passed to another classifier consisting of diverse layers that learn the complexity of domain-specific features. A probabilistic model, such as sigmoid or softmax, is then applied to the resulting output. Our proposed BERT model was fine-tuned for virology-related research, enabling it to acquire a more sophisticated understanding of nucleotides, amino acids, and proteins. Once the input context score vector had been obtained, it was fed into a softmax probabilistic layer, denoted as P, as expressed by Equation (1).

(1) $$P=Softmax\left(CW^{T}+b^{T}\right)$$

The general softmax Equation (2) was used to compute the probability distribution of the output class, while the Categorical Cross Entropy loss function (3) was employed to measure the dissimilarity between the predicted class probabilities and the true class label.

(2) $$\alpha {\left(z\right)}_{i=}=\frac{e^{z_{i}}}{\sum_{j=1}^{K}e^{z_{j}}}$$

(3) $$L_{CE}=-\sum_{i=1}^{n}t_{i}\log\left(p_{i}\right),for\;n\;Classes$$

Here, as t represents the true label at time as i and as p represents the softmax probability for the jth class at time i.

### 3.4. Evaluation Metrics

A wide range of matrices are used for evaluating machine learning models. These parameters help us to measure the efficiency of the generated model. The evaluation of a model is based on True Positive (TP), True Negative (TN), False Positive (FP), and False Negative (FN). The confusion matrix provides a comprehensive evaluation of the model’s performance, allowing for further analysis and potential improvements in the classification process. Based on the confusion matrix, we calculated the accuracy and F-score. Accuracy is used to evaluate the model’s performance. Accuracy measures the number of correct predictions made over the entire test dataset.

(4) $$Accuracy=\frac{TP+FP}{TP+FP+TN+FN}$$

The F-score is a weighted average of precision and recall, and it is used to evaluate the performance of a classification model. Thus, to calculate the F-score we need to calculate the precision and recall.

Precision P is the proportion of TP out of all predicted positives (TP+FP).

(5) $$P=\frac{TP}{TP+FP}$$

Recall (R) is the proportion of true positives (TP) out of all actual positives (TP+FN).

(6) $$R=\frac{TP}{TP+FN}$$

F−score  is the harmonic mean of precision and recall, that is:

(7) $$F-score\;=2*\;\frac{p*R}{P+R}$$

The pseudo-code of the proposed model is described below in Table 2.

## 4. Results and Discussion

The experiment conducted involved selecting a certain amount of data, shown in Table 3, which lists several diseases along with their corresponding counts representing the number of cases or occurrences of each disease. The table shows that the counts range from a high of 5000 to a low of 28, indicating the relative prevalence of each disease.

The BERT model was configured with different parameters, as shown in Table 4. The model specified with 2 layers, each with 2 attention heads and 768 hidden units per layer, can handle input sequences of up to 5000 tokens in length and is designed to handle inputs consisting of a single segment. The optimizer was set up to train the model using the Adam optimizer with a small value of epsilon to avoid division by zero when computing the optimizer’s update step. The argument “freeze_bert” was set to False, indicating that the parameters of the pre-trained BERT model will be updated during training.

The maximum sequence length of the BERT model was set to 5000, meaning that any input sequence longer than 5000 tokens would be truncated. This experiment’s choice of using the Adam optimizer is suitable for models with a large number of parameters, such as BERT.

**Figure 3:**
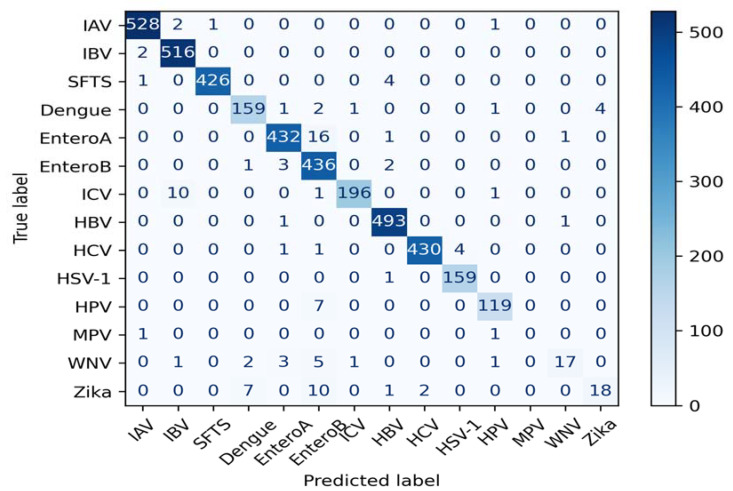
Results of confusion matrix.

The evaluation of the proposed multi-class classification model was carried out using a confusion matrix, as illustrated in Figure 3. The 14 different classes of viruses considered in the model were *IAV*, *IBV*, *SFTS*, *Dengue*, *EnteroA*, *EnteroB*, *ICV*, *HBV*, *HCV*, *HSV-1*, *HPV*, *MPV*, *WNV*, and *Zika*. This confusion matrix showed the performance of a multi-class classification model on a set of test data. The matrix was structured in such a way that the rows indicate the true classes of viruses, while the columns represent the predicted classes. The numbers in the cells of the matrix represent the number of instances that were either correctly or incorrectly classified by the model. For instance, the cell in the first row and second column indicates that there were two instances of the *IAV* virus that were incorrectly predicted to be *IBV* by the model. Similarly, the cell in the fourth row and fifth column shows that one instance of the Dengue virus was incorrectly predicted to be *EnteroA* by the model.

To analyze the performance of the model more closely, we can calculate various evaluation metrics such as precision, recall, and F1 score. Based on the given confusion matrix, the results obtained from the experiment were an accuracy and F-Score of 96.47 and 93.46, respectively. A high F-score indicates that the model has a high accuracy and is successful at identifying true positives while minimizing false positives and false negatives.

The confusion matrix presented a clear representation of the model’s performance in terms of accuracy and F-Score. Looking at the matrix, we can see that the model’s performance was generally good. The majority of instances have been correctly classified, and most of the off-diagonal entries are small.

**Figure 4:**
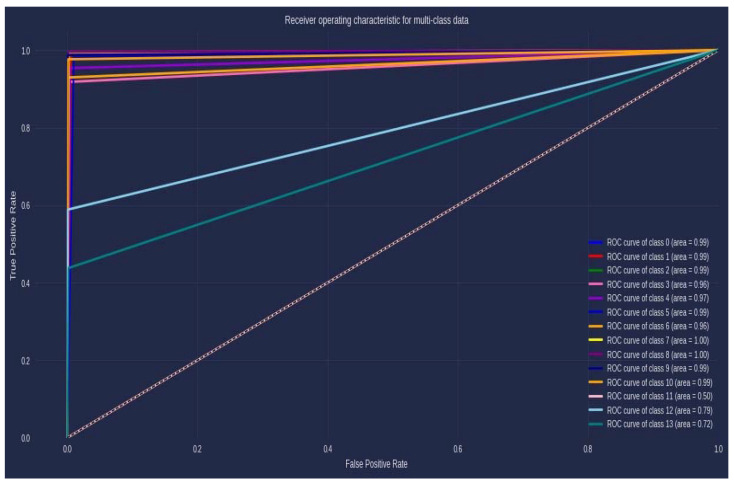
ROC Curve.

The ROC curve, as presented in Figure 4, provides a way to evaluate the performance of a proposed model. In the ROC curve, each virus is labeled from 0 to 13, respectively, as *IAV*, *IBV*, *SFTS*, *Dengue*, *EnteroA*, *EnteroB*, *ICV*, *HBV*, *HCV*, *HSV-1*, *HPV*, *MPV*, *WNV*, and *Zika* virus. According to the ROC plot, each class (i.e., virus) has a corresponding AUC value. Classes 0, 1, 2, 5, 9, and 10 have an AUC value of 0.99. Classes 3 and 6 have an AUC value of 0.96. Classes 7 and 8 have an AUC value of 1.00, which indicates perfect performance. Similarly, classes 11, 12, and 13 have AUC values of 0.50, 0.79, and 0.72, respectively.

The proposed method was rigorously compared with various techniques to demonstrate its robustness, as presented in Table 5. The model utilized the BERT architecture for DNA analysis, resulting in a remarkable accuracy of 97.69%. In comparison, the model presented in [23] used the BiLSTM model to classify the DNA sequences of MPV and HPV viruses and achieved an accuracy of 96.08%. The authors in [8] employed a CNN for DNA sequence classification and achieved an accuracy of 93.16%. Similarly, the XGboost algorithm was used to classify five types of chromosomes, resulting in an accuracy of 89.51% [24]. These results demonstrate the superior performance of the proposed method over existing techniques in genomic data analysis.

## 5. Conclusions

In conclusion, this research work successfully applied a specialized BERT tokenizer and architecture designed for DNA analysis to analyze viral genomic data. The proposed system consisted of a nucleotide acquisition pipeline and a customized BERT model for genomic data analysis. The study collected nucleotide sequences from various viruses, including *Zika*, *influenza*, *HPV*, *WNA*, *hepatitis*, *dengue*, and others from GenBank, and employed advanced data balancing techniques to address any potential data imbalance. The BERT architecture was customized for DNA analysis, and a classifier was used to identify important features to understand the relationship between genotype and phenotype. The proposed approach achieved an impressive accuracy of 97.69%.

While the study focused on a wide range of viruses, there are still many other viral species that could be analyzed using the proposed system in the future. Investigating the performance of the BERT architecture on a broader range of viruses may provide valuable insights, which will be considered in future research.

## Figures and Tables

**Table 1:** Taxonomic levels of viruses.

Order	Family	Genus	Species
Articulavirales	Orthomyxoviridae	*Alphainfluenzavirus*	*IAV*
Articulavirales	Orthomyxoviridae	*Betainfluenzavirus*	*IBV*
Articulavirales	Orthomyxoviridae	*Gammainfluenzavirus*	*ICV*
Bunyavirales	Phenuiviridae	*Bandavirus*	*SFTS*
Flaviviridae	Flaviviridae	*Flavivirus*	*Dengue*
Picornavirales	Picornaviridae	*Enterovirus*	*Enterovirus A*
Picornavirales	Picornaviridae	*Enterovirus*	*Enterovirus B*
Blubervirales	Hepadnaviridae	*Orthohepadnavirus*	*HBV*
Amarillovirales	Flaviviridae	*Hepacivirus*	*HCV*
Herpesvirales	Herpesviridae	*Human alphaherpesvirus 1*	*HSV-1*
Zurhausenvirale	Papillomaviridae	*Alphapapillomavirus*	*HPV*
Chitovirales	Poxviridae	*Orthopoxvirus*	*MPV*
Amarillovirales	Flaviviridae	*Flavivirus*	*WNV*
Amarillovirales	Flaviviridae	*Flavivirus*	*Zika*

**Table 2:** Pseduo-code.

Input: NCBI GenBank nucleotide sequences
Output: Biomarkers extracted from genome
Let us denote the set of input nucleotide sequences as S, and the set of extracted biomarkers as B. Here is the mathematical representation of the given pseudo-code:
● Collect nucleotide sequences:
● S = {s1, s2, …, sn}
● Filter and screen the sequences:
● S′ = {s|s meets certain criteria}
● Transform the genomes into k-mers:
● K = {k1, k2, …, km}, where ki is a k-mer of a nucleotide sequence s
● Train the BERT tokenizer on the k-mers:
● T = Tokenizer.train (K)
● Use SMOTE to balance imbalanced genomic data samples:
● S″ = SMOTE (S′)
● Perform additional preprocessing steps for the BERT model:
● Convert nucleotide sequences to DNA-specific tokens using T
● Apply necessary transformations to prepare the data for the BERT model
● Preprocess the nucleotide sequence for the custom BERT model:
● Tokenize the nucleotide sequence using the proposed DNA/RNA tokenizer
● Pad any gaps or missing sequence regions with specific tokens
● Encode the input k-mers into a bidirectional representation using the BERT model’s bidirectional encoder:
● E = Encoder.encode (S″)
● Extract specific biomarkers from the genome in an unsupervised manner using the BERT model:
● B = Biomarker.extract (E)
● Pass these biomarkers into a deep neural network-based classifier:
● Classifier.train (B)

**Table 3:** Number of occurrences of each disease.

Disease Name	Count	Disease Name	Count
*HBV*	5000	*Gamma Influenza Virus*	1941
*Betta Influenza Virus*	5000	*Dengue*	1866
*Alpha Influenza Virus*	5000	*Human Alpha Herpes*	1479
*Entero Virus B*	4653	*Human Papilloma Virus*	1355
*Hepaci Virus*	4619	*West Nile Virus*	371
*Entero Virus A*	4527	*Zika Virus*	321
*Dabie Banda Virus*	4193	*Monkey Pox*	28

**Table 4:** BERT model configuration.

**Parameter Name**	**Details**	**Parameter Name**	**Details**
Maximum position embeddings	5000	Number of hidden layers	2
Number of attention heads	2	Hidden size	768
Training ratio	80	Testing ratio	20
Freeze_bert	False	epsilon value	0.00000001
Learning Rate	0.00005	optimizer	Adam

**Table 5:** Comparison with some recent models.

Ref	Year	Method	Accuracy (%)
Proposed	-	BERT Architecture	97.69
[23]	2022	BiLSTM model	96.08
[8]	2021	CNN model	93.16
[24]	2020	XGboost algorithm	89.51

## Data Availability

https://ftp.ncbi.nih.gov/genbank/ (accessed on 27 February 2023).
